# Evaluating the Effectiveness of a Proposed 11-Step Community-Based Interventional Program That Educates, Assesses Risk, and Overcomes Barriers to Complete Screening Colonoscopy Among African Americans

**DOI:** 10.7759/cureus.112817

**Published:** 2026-07-16

**Authors:** Deepika Mannem, Sufia Ahmed, Wei Wei, Eileen Meisler, Eberechi Agwa, Carol Burke, Samir Abraksia

**Affiliations:** 1 Internal Medicine, Cleveland Clinic, Cleveland, USA; 2 Lerner Research Institute (LRI) Quantitative Health Sciences, Cleveland Clinic, Cleveland, USA; 3 Taussig Cancer Institute, Cleveland Clinic, Cleveland, USA; 4 Hematology and Medical Oncology, Cleveland Clinic, Cleveland, USA; 5 Gastroenterology, Cleveland Clinic, Cleveland, USA

**Keywords:** barriers to healthcare, cancer screening, colorectal cancer, community participation, educational program, risk assessment

## Abstract

Objective

This study evaluated an 11-step Community-Based Interventional Program (CBIP) designed to improve colorectal cancer (CRC) knowledge, assess perceived CRC risk, and identify barriers to CRC screening among African Americans.

Methods

CBIPs were conducted at multiple community locations and included a 30-minute educational presentation on CRC screening. Participants completed pre- and post-education surveys assessing CRC knowledge, demographics, CRC risk, and barriers to screening. Program effectiveness was evaluated using participant satisfaction ratings.

Results

A total of 166 participants attended sessions of 10-20 individuals. Median correct responses increased from eight (pre-test) to nine (post-test) (p = 0.52). CRC risk was average in 40.4% of participants and above average in 59.6%. Common barriers included the need for appointment reminder calls (14.3%), transportation (13.5%), and an accompanying adult (12.1%). Seventy percent preferred receiving education before screening, and 84.3% reported satisfaction with the program.

Conclusion

This CBIP was well-received and identified key barriers to CRC screening. Although limitations exist, this feasibility study can serve as a guide for further research into community-based interventions.

## Introduction

Colorectal cancer (CRC) is the third most common cancer in African American men and women and is the second leading cause of cancer-related death [1,2]. African Americans exhibit the highest CRC mortality of all US populations [3,4]. African Americans, when compared to Caucasians, have lower CRC screening utilization and an earlier presentation of CRC (0-8 years younger than Caucasians). Several studies and systematic reviews demonstrate that African American patients are more likely to have tumors with molecular and clinicopathologic features associated with worse prognosis and treatment resistance [5]. Statistically significant differences in overall survival (OS) among racial and ethnic groups were observed for all stages (P < 0.001, P = 0.04, and P < 0.001 for stages 1 and 2, stage 3, and stage 4, respectively), but the magnitude of difference was greatest among patients with stage 4 disease, similar to that which has been observed in the US national registry data. For patients with stage 4 disease, median OS was 64, 32, 45, and 40 months for Asian, Black, Hispanic, and White patients, respectively [6]. Medical literature supports that African Americans with colorectal cancer are more likely to have right-sided microsatellite-stable and EMAST-positive tumors, all of which are unfavorable prognostic features and contribute to observed disparities in treatment outcomes compared to Caucasian patients [7]. Literature also supports that many African Americans possess gut microbiomes more conducive to initiating and/or propagating colonic neoplasia than Caucasians [8]. These epidemiological, biological, and genetic parameters put African Americans at higher risk of CRC irrespective of socioeconomic barriers in the community.

The majority of our patients at Cleveland Clinic South Pointe Hospital come from Cuyahoga County. The 2025 Cuyahoga County Census showed that the population comprised 61.7% "White alone" and 26.7% "Black alone"; however, the population treated at South Pointe Hospital is primarily African American [9]. This region in Cuyahoga County accounts for an 18.5% higher poverty rate than Ohio's overall poverty rate and has a higher percentage of residents aged 25 and older without a high school diploma compared to the Ohio average [10]. The Ohio Department of Health notes increased incidence and mortality from several types of cancer in this community when compared to the rest of Ohio. A 2019 community needs assessment identified that our region needs access to affordable healthcare, chronic disease management, and preventative medicine [11,12]. These disparities highlight the need for programs that improve cancer screening, as well as patient navigation programs to assist with overcoming barriers to obtaining health screenings.

Barriers to CRC screening fall into four categories: attitudes and beliefs, healthcare and healthcare system barriers, cost, and others [12-15]. Clinical risk factors for CRC include age, family history, obesity, diabetes, alcohol use, and long-term smoking [16,17].

## Materials and methods

Study design

**Figure 1:**
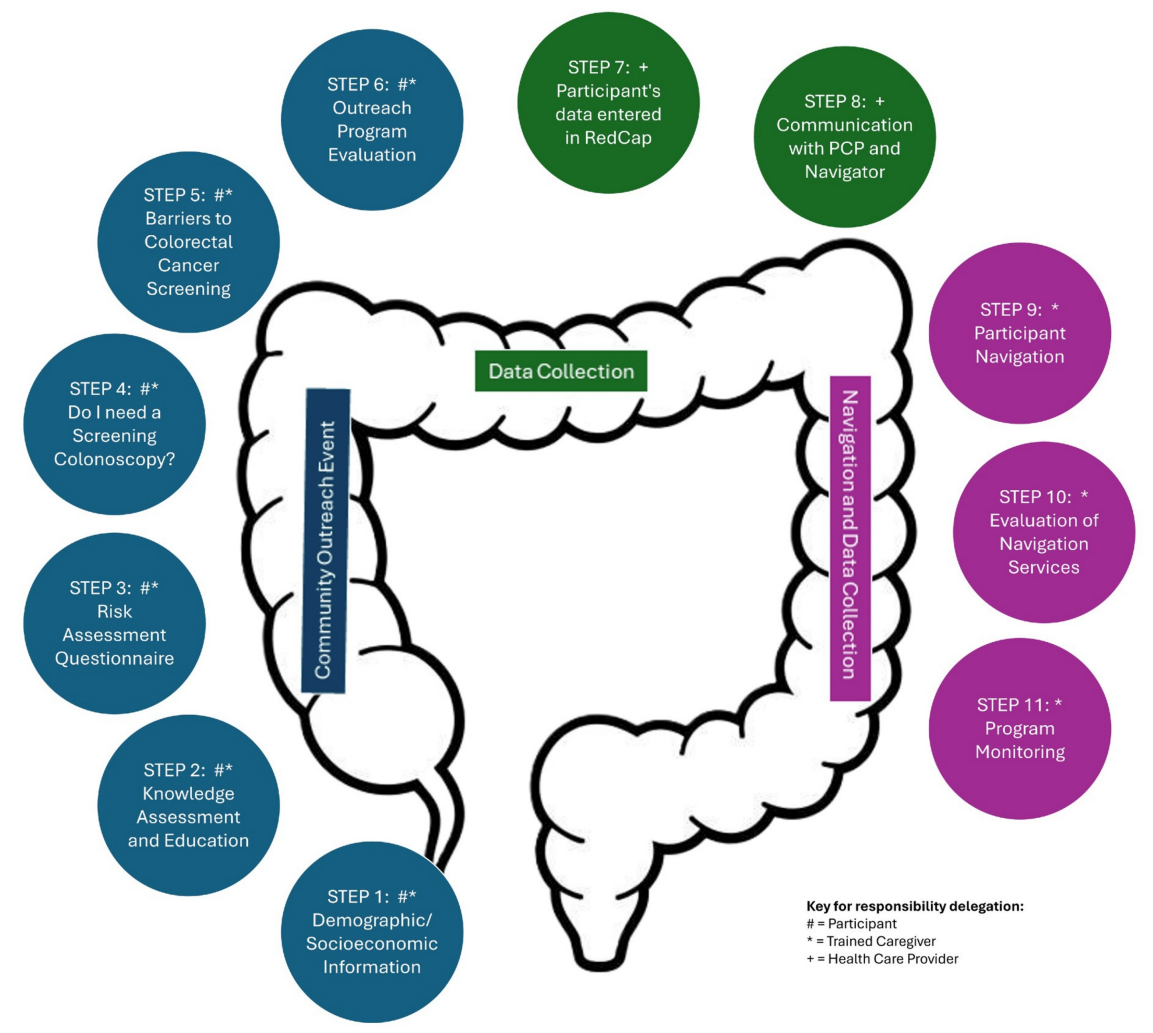
Diagram of 11-step Community-Based Interventional Program Image created using Microsoft PowerPoint (Microsoft Corp., Redmond, WA) PCP: primary care provider

The study was designed as a multistep community-based outreach program to educate participants about CRC screening options, assess risk, identify barriers to screening, and assess satisfaction with the education program [18]. Steps 1-6 are accomplished via a community outreach program; steps 7-8 entail data collection and communication with primary care providers (PCP) or gastroenterologists (GI) if needed; and steps 9-11 relate to the evaluation of navigation and program monitoring (Figure 1). The design intends to create a community outreach program that clearly defines each provider's responsibilities, specifies timeframes for the completion of study tasks, and engages primary care providers. Systematic reviews did not fully address these issues.

**Figure 2:**
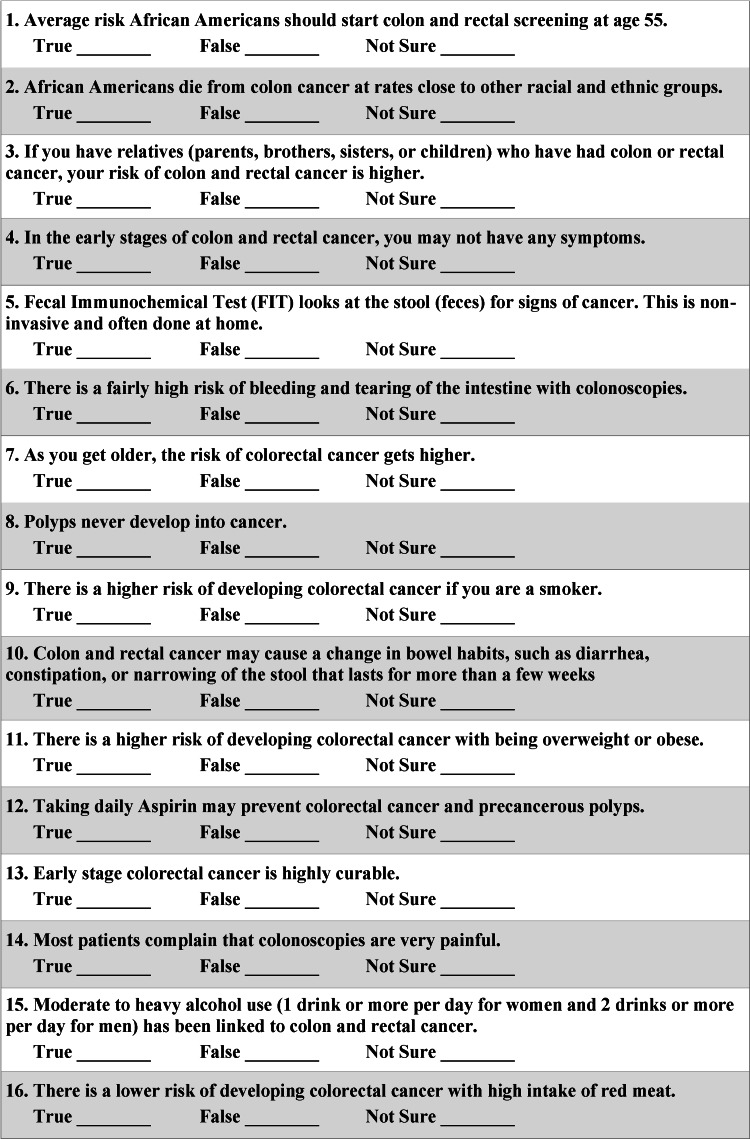
Sixteen-item questionnaire provided in participant packet

Community-Based Interventional Program (CBIP) or outreach events were held at churches and community centers in the local area over multiple days. Members of the community and churchgoers were invited to attend a 30-minute educational PowerPoint presentation on colorectal cancer screening. The facilitators of the education session were a clinical research coordinator in the department of hematology/oncology, a community oncologist, and internal medicine residents. This was a one-time event for each participant. Data collection packets were made and distributed to participants at the community outreach events. This packet included a demographic survey and a pre-education questionnaire, both completed at the beginning of the event. The 16-item questionnaire (Figure 2) and the educational session covered colorectal cancer signs and symptoms, risk factors, screening, and follow-up. The 16-item questionnaire was developed specifically for this CBIP. Following the education session, participants completed the post-education questionnaire with the same 16 questions as the pre-education questionnaire but in a different order. The pre- and post-test questionnaires included the options "true," "false," and "not sure" as possible answers participants could select for each statement. For the statistical analysis, the "not sure" option and unanswered questions were counted as incorrect answers. Regarding the most common barriers, these were reported as percentages that were calculated as the number of participants who answered "yes" to each barrier out of the total number of participants who answered each question. A satisfaction survey was also included in the packet. The packets were collected at the end of each event, and all data were recorded and stored in an Institutional Review Board (IRB)-approved, secure database (REDCap, Vanderbilt University, Nashville, TN).

Inclusion and exclusion criteria

This study analyzed data from African American participants only. Exclusion criteria included individuals under the age of 18 and prior participants. We utilized the United States Preventive Services Task Force (USPSTF) recommendations for screening at 45 years of age [19]. Sample size calculations were conducted using Power Analysis and Sample Size (PASS) software version 15 (NCSS, LLC, Kaysville, UT) to achieve >0.9 power and determined that 150 participants were needed. This study was approved by the Cleveland Clinic IRB (approval number: 18-855) and supported by the Cleveland Clinic Taussig Cancer Institute Outreach Program and a foundation grant from VeloSano. Recruitment and data collection for this study occurred from March 2019 to December 2024, with recruitment being suspended from January 2020 to December 2021.

Data collection

The primary endpoint is the total knowledge score from the pre- and post-session questionnaires. Pre-education and post-education scores were summarized as median, interquartile range (IQR), and range. The accuracy status of pre- and post-education was summarized in frequencies and percentages for each question.

Statistical analysis

The Wilcoxon signed-rank test was used to compare total knowledge scores pre- and post-education. Patients were included in the primary analysis if they completed both the pre-test and post-test. Secondary endpoints included risk assessment and the identification of barriers. Individual questions were tabulated and compared between pre- and post-tests as secondary analyses. The secondary endpoints of risk assessment (average risk or high risk) and barriers were summarized in frequencies and percentages. The barriers to obtaining colonoscopy were ranked in order from most commonly to least commonly identified barriers as secondary endpoints.

## Results

A total of 166 participants out of 202 who attended and participated in the events were included in the final analysis. Characteristics of participants are outlined in Table 1. Ninety-four percent of attendees identified as African American.

**Table 1:** Demographic information of participants in the study N, number of participants; %, calculated out of 166 participants included in the analysis; PCP, primary care provider; GED, general equivalency diploma

Sex	N	%
Not answered	7	4.22
Male	35	21.08
Female	124	74.7
Marital status		
Not answered	13	7.83
Single	55	33.13
Married	27	16.27
Divorced	27	16.27
Widow	40	24.1
Partner	3	1.81
Separated	1	0.6
Occupation		
Not answered	9	5.42
Employed, working 40 or more hours per week	28	16.87
Employed, working 1-39 hours per week	9	5.42
Not employed, looking for work	4	2.41
Not employed, not looking for work	10	6.02
Retired	73	43.98
Disabled, not able to work	33	19.88
Are you currently being followed by a PCP?		
Not answered	26	15.66
No	13	7.83
Yes	127	76.51
Do you have health insurance?		
Not answered	13	7.83
No	2	1.2
Yes	151	90.96
How long did you go to school?		
Not answered	11	6.63
Some high school	17	10.24
High school diploma/GED	42	25.3
Some college	57	34.34
College graduate	28	16.87
Postgraduate degree	11	6.63
What is your ethnicity? (choice = Hispanic)		
Unchecked	164	98.8
Checked	2	1.2
What is your ethnicity? (choice = White)		
Unchecked	163	98.19
Checked	3	1.81
What is your ethnicity? (choice = Black/African American)		
Unchecked	10	6.02
Checked	156	93.98

The knowledge scores are depicted in Table 2. The median total knowledge score on the pre-test was 8/16 (range: 0-16) and 9/16 (range: 0-13) on the post-test survey (p-value: 0.52).

**Table 2:** Primary endpoint of total knowledge scores pre-test and post-test and paired comparison results The Wilcoxon signed-rank test was used to compare total knowledge scores pre- and post-education N, number of participants; NA, not available

	N	Minimum	25th percentile	Median	75th percentile	Maximum	P-value
Total knowledge score, pre-test	166	0	5	8	11	16	NA
Total knowledge score, post-test	166	0	7	9	10	13	NA
Knowledge score change (post-pre)	166	-9	-3	0	2	9	0.52

Performance on individual questionnaire items and the comparison of each from pre- to post-test are outlined in Table 3. For questions 2, 4, 5, 8, 10, and 14, more than half of the participants changed their correct answers in the pre-test to incorrect answers in the post-test. For questions 3, 6, 9, 11, 12, and 13, more than half of the participants changed their incorrect answers in the pre-test to correct answers in the post-test (Table 3).

**Table 3:** Percentages of participants who changed their answer from incorrect to correct response and vice versa when comparing the results of the pre- and post-test FIT: fecal immunochemical test

Item number	Item	Percentage participants changed answer from incorrect to correct (better)	Percentage participants changed answer from correct to incorrect (worse)
1	Average-risk African Americans should start colon and rectal screening at age 55	32.38	45.9
2	African Americans die from colorectal cancer at rates close to other racial and ethnic groups	6.56	93.18
3	If you have relatives (parents, brothers, sisters, or children) who have had colon or rectal cancer, your risk of colorectal cancer is higher	62.5	18.63
4	In the early stages of colorectal cancer, you may not have any symptoms	15.38	84.16
5	I completed a FIT test this year, so I do not need to complete one next year	6.74	96.1
6	There is a fairly high risk of bleeding and tearing of the intestine with colonoscopies.	63.64	12.82
7	Polyps never develop into cancer	40.38	22.81
8	There is a higher risk of developing colorectal cancer if you are a smoker	11.39	85.06
9	If my FIT test result is not normal, I will need a colonoscopy	80.25	10.59
10	Colorectal cancer may cause a change in bowel habits, such as diarrhea, constipation, or narrowing of the stool that lasts for more than a few weeks	26.53	65.81
11	There is a higher risk of developing colorectal cancer with being overweight or obese	63.75	8.14
12	Taking daily aspirin may prevent colorectal cancer and precancerous polyps	79.43	16
13	Early-stage colorectal cancer is highly curable	76.74	14.63
14	Most patients complain that colonoscopies are very painful	4	89.01
15	Moderate to heavy alcohol use (one drink or more per day for women and two drinks or more per day for men) has been linked to colorectal cancer	40	31.37
16	There is a lower risk of developing colorectal cancer with a high intake of red meat	46.73	32.2

Secondary endpoints included risk assessment and the identification of barriers. Regarding risk assessment, 63 participants (40.4%) identified as average risk and 93 (59.62%) identified as high risk for CRC (Table 3). Of those who identified as high risk, fewer than half (40%) indicated that they had an established PCP or GI doctor (Table 4).

**Table 4:** Risk assessment of study participants and the percentage of high-risk participants who indicated whether they do or do not have a PCP or GI doctor N, number of participants; %, calculated out of 166 participants included in the analysis; PCP, primary care provider; GI, gastroenterologist

Total risk score	N	%
Not answered	9	5.42
0	65	39.16
1	50	30.12
2	28	16.87
3	9	5.42
4	5	3.01
Average risk (0 total score)		
Not answered	10	6.02
No	93	56.02
Yes	63	37.95
Higher risk (more than 0 total score)		
Not answered	11	6.63
No	62	37.35
Yes	93	56.02
If you are at a higher risk (risk score: ≥1), do you have a PCP and/or GI doctor?		
Not answered	79	47.59
No	20	12.05
Yes	67	40.36
If you are at a higher risk (risk score: ≥1), are you being followed by a PCP and/or GI doctor?		
Not answered	98	59.04
No	19	11.45
Yes	49	29.52

The most common barriers identified by participants in the survey are reported in Table 5. They are as follows: I need a reminder call for the appointment (14.3%), I need transportation (13.5%), I need someone to accompany me (12.1%), I need a primary care physician referral (11.4%), I need appointment coordination (10.6%), I need financial assistance (5.88%), and I need education about colonoscopy preparation and procedure (4.95%) (Table 5). Notably, a number of participants left each item blank in this portion of the survey; unanswered items were left out of the statistical analysis for this portion.

**Table 5:** Barriers to obtaining colonoscopy N, number of participants; %, calculated out of 166 participants included in the analysis

Barriers to obtaining colonoscopy	N	%
1. I need a primary care physician referral		
No	93	88.6
Yes	12	11.4
2. I need transportation		
No	91	87.5
Yes	14	13.5
3. I need appointment coordination		
No	93	89.4
Yes	11	10.6
4. I need financial assistance		
No	96	94.1
Yes	6	5.88
5. I need a reminder call for appointment		
No	90	85.7
Yes	15	14.3
6. I need education about colonoscopy preparation and procedure		
No	96	95.05
Yes	5	4.95
7. I need someone to accompany me		
No	94	87.6
Yes	13	12.1

According to the post-program satisfaction survey, 69.9% of participants reported that they would prefer to have this type of education before having CRC screening. Overall, 84.3% reported that they were satisfied with the program (selected "strongly agree" or "agree" to the following statement: "Overall, I am satisfied with the program") (Table 6).

**Table 6:** Results of satisfaction survey

Results of satisfaction survey	N	%
1. I would prefer to have this type of education before having colorectal cancer screening		
Not answered	38	22.89
No	12	7.23
Yes	116	69.88
2. I will likely choose the lifestyle recommendations provided to me today		
Not answered	34	20.48
Strongly agree	80	48.19
Agree	48	28.92
Neutral	2	1.2
Strongly disagree	2	1.2
3. I found risk questionnaire helpful		
Not answered	29	17.47
Strongly agree	89	53.61
Agree	47	28.31
Neutral	1	0.6
4. I found that attending this type of program is convenient (suitable and with minimal disruption to my lifestyle)		
Not answered	31	18.67
Strongly agree	88	53.01
Agree	44	26.51
Neutral	3	1.81
5. I found that this program is helpful in addressing an important health problem		
Not answered	26	15.66
Strongly agree	102	61.45
Agree	36	21.69
Neutral	2	1.2
6. Overall, I am satisfied with the program		
Not answered	25	15.06
Strongly agree	96	57.83
Agree	44	26.51
Neutral	1	0.6
7. The program timing is just right		
Not answered	27	16.27
Strongly agree	87	52.41
Agree	49	29.52
Neutral	2	1.2
Disagree	1	0.6

## Discussion

The purpose of the study was to assess the feasibility of a community-based intervention to educate, assess risk, and identify barriers to obtaining colorectal cancer screening in a predominantly African American community. In this study, data were collected to evaluate the efficacy of the education program through pre-test and post-test questionnaires, assess individual risk for CRC, and identify barriers to CRC screening. The incidence and impact of colorectal cancer can be significantly reduced by implementing primary prevention strategies such as adopting a healthy lifestyle, avoiding risk factors, and practicing early detection through screening [20]. This task is very difficult to achieve in the outpatient settings; as presented by Oxentenko et al., there are tangible opportunities to improve the CRC screening process in primary care residency programs [14].

Performance on specific pre-test items was examined to assess areas of knowledge deficiency regarding colon cancer screening prior to the educational presentation. More than half of the participants got pre-test items 1, 2, 5, 6, 12, 15, and 16 incorrect (including unanswered and "not sure" as incorrect answers) (Figure 2). This highlights potential areas of knowledge deficiency among the population studied that future PowerPoints should address to correct misconceptions regarding colon cancer screening, signs, and symptoms. As more than half of the participants either were unsure of the answers to these questions or answered these questions incorrectly, the future educational sessions must explicitly address these topics to improve knowledge in these areas among the population studied.

When comparing the results of the pre- to post-test, the median knowledge improvement from pre- to post-test was 0 (p-value: 0.52), indicating no significant improvement in knowledge. The analysis of performance on individual items revealed limitations of the educational intervention in improving knowledge in certain areas; more than half of the participants changed their correct answer on these questions on the pre-test questionnaire to an incorrect answer post-test (Table 3). There were six items on which patients changed their answers from correct on the pre-test to incorrect on the post-test, indicating that these are the areas that future education should focus on emphasizing. We proposed areas of improvement, as more than 65% of the participants answered the question incorrectly. Questions 2, 4, 5, 8, 10, and 14 (Table 3) are topics that need to be improved in the education session, and we intend to stress these concepts in future education sessions. We may need to explicitly address these topics in a clear, easy-to-understand manner. For example, the slides dedicated to addressing symptoms of colon cancer should explicitly state that in the early stages of colon cancer, there may not be any symptoms present and that colorectal cancer may cause a change in bowel habits that lasts longer than a few weeks.

Additional concepts may need to be addressed in future educational programs. When looking at the questions, we need to understand our participant population and see if the questions are written clearly and if they are easy to understand. There are many reasons a participant could have changed their answer, including, but not limited to, the method of education, information provided in the education session, or even the speed of the presentation. Everyone learns in different ways, and this could mean that we need to expand the way we do our teaching in future programs. One possibility for future education could be an interactive tablet where people can click through the educational content at their own pace.

On the other hand, on questions 3, 6, 9, 11, 12, and 13, more than half of the participants changed their incorrect answer in the pre-test to a correct answer in the post-test (Table 3). This indicates that the educational session was effective in addressing these topics on individual risk with family history, risks of colonoscopy, how fecal immunochemical test (FIT) tests are used, how to prevent colon cancer, and the curability of colon cancer. As the majority of participants demonstrated knowledge improvement in these areas from pre- to post-test, we believe the educational session was effective at addressing these topics. These are important educational points, so we can continue to include them as they were presented in the program.

Regarding assessing risk, more than half of the participants (59.62%) who answered the risk questionnaire were identified as having a high risk of developing colon cancer (Table 4). This assessment clearly highlights the importance of earlier colon cancer screening and preventative outreach programs. This knowledge can help raise awareness and empower patients to take charge of their own health by prioritizing early detection and taking proactive measures to reduce their risk. Our data validate the need to start colorectal cancer screening at a younger age (40 years old) among this high-risk population, screen in appropriate intervals, and encourage the adoption of a healthy lifestyle.

A systematic review by Wang et al. found that major barriers to obtaining colorectal cancer screening are essentially not different between rural and urban populations [21]. The barriers included in the study were the fear or embarrassment of the colonoscopy procedure, fear of finding cancer, lack of recommendation to get a colonoscopy by a physician, lack of knowledge, and lack of perceived need to obtain screening [21].

Our study was conducted in a suburban setting, and we were able to identify barriers to CRC screening. The top barriers were identified as needing a reminder call for the appointment, transportation, and needing someone to accompany participants to the appointment. Our survey included seven possible barriers (Table 5). Our top barriers differed from the systematic review by Wang et al., which shows that every population will have different barriers that are unique and need to be investigated and addressed [21].

A unique consideration with colonoscopy screening is getting home after the procedure, since the procedural sedation and anesthesia given will preclude patients from driving afterward. Strategies to overcome barriers to obtaining colonoscopy as identified by the survey include establishing patient navigation programs to call patients with reminders for their appointment and assist patients with transportation to and from their appointment. Along the same lines, several patients indicated that they needed someone to accompany them to the appointment. This barrier can be overcome by establishing a program in which dedicated patient navigators accompany patients to their colonoscopy appointments. These are potential areas to address in the effort to educate, raise awareness, and improve adherence.

Additionally, the majority of participants responded positively to the post-program satisfaction survey (Table 6), indicating that education on preventive colon cancer screenings is well-received by the community as a viable method to raise awareness.

Among all racial groups, African Americans rank last in colon cancer screening rates, colorectal cancer mortality, and five-year survival rates [22].A cross-sectional survey of an African American population found that sociocultural attitudes and the lack of knowledge about colorectal cancer screening were factors that prevented participants from seeking out screening [19]. Participants in the cross-sectional study felt that having an in-depth conversation with their primary care provider was a critical step in obtaining screening [22]. Likewise, a meta-analysis found that an individual's decision to obtain colorectal cancer screening depends on awareness [17]. The awareness of colorectal cancer screening modalities can influence motivation to obtain screening. Education sessions to correct the public's misconceptions and primary care physicians' efforts to recommend screening are two factors that can improve awareness around CRC screening [17].

A combination of individual provider and patient factors, as well as the institutionalized racism that permeates the healthcare system, influences compliance with colorectal cancer screening among African Americans [23]. Examples of institutionalized racism include a lack of cultural representation in educational materials and perceptions of inadequate or unequal care [23,24]. To address these barriers, open communication and building strong interpersonal relationships between patient and physician can establish the trust needed to improve knowledge about and compliance with colorectal cancer screenings.

Following the educational session, steps 7-8 and 9-11 seen in Figure 1 were completed by our patient navigators within the Cleveland Clinic Taussig Cancer Center. There were many obstacles to the navigation process after the educational session, including the inability to contact the participant, patients getting care at a different hospital system, in which we could not access the data, and even being lost to follow-up.

Our study had several limitations, including the sample size. It is possible that with a large population, we would have been able to assess whether the education portion of the intervention program was adequate. Our study was also conducted in a very small region of Cuyahoga County, so these data cannot be generalized to the public. Our questionnaires and surveys were conducted with handwritten paper and pen. With the physical packets, some participants left fields or questions blank, omitting crucial information, including contact details for post-education navigation. A possible solution could include doing the questionnaire on an electronic device, such as a tablet. This way, the participants must complete each section prior to moving on to the next page.

Our study aimed to raise awareness and identify specific areas of knowledge deficiency about colorectal cancer screening through the questionnaire and address these misconceptions through the educational session. Future studies can work to analyze compliance after the educational session and can share results from pathology with participants and refer to general surgery, genetic counseling, medical oncology, or other necessary specialties. We have realized that follow-up may be difficult because we have different electronic medical records (EMRs) using a different hospital system in the city of Cleveland.

Also, our project could be incorporated into health fairs or preventative medicine presentations, as they are seen as a great avenue to spread awareness [25]. This presentation could also be expanded to other areas in the community, including hair salons, senior centers, and fitness centers, to reach a wider, more diverse population. In future trials, the educational session should be improved using our results to accommodate the gaps in the knowledge of the population we serve. The goal would be to change the community's perception of screening, spark interest, and essentially reduce barriers.

## Conclusions

In conclusion, this 11-step program was well-received by our underserved, predominantly African American, community. Most of the participants were satisfied with the educational component. We were able to identify barriers that need to be addressed and assess the risk for colorectal cancer. Although our study had limitations, this feasibility study can be used as a guide for future educational programs, as this is an area that requires further research.

## Figures and Tables

**Figure 3:**
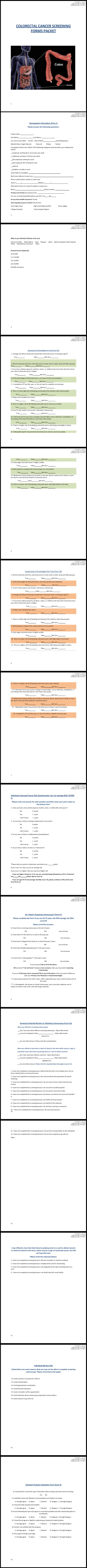
Participant packet PCP, primary care provider; GI, gastroenterologist

## Appendices

Figure 3 shows the participant packet.
